# Correction: Absolute standard hydrogen electrode potential and redox potentials of atoms and molecules: machine learning aided first principles calculations

**DOI:** 10.1039/d5sc90103k

**Published:** 2025-05-21

**Authors:** Ryosuke Jinnouchi, Ferenc Karsai, Georg Kresse

**Affiliations:** a Toyota Central R&D Labs., Inc. Yokomichi 41-1 Nagakute Aichi Japan jryosuke@mosk.tytlabs.co.jp; b VASP Software GmbH Berggasse 21 A-1090 Vienna Austria; c University of Vienna, Faculty of Physics Kolingasse 14-16 A-1090 Vienna Austria

## Abstract

Correction for ‘Absolute standard hydrogen electrode potential and redox potentials of atoms and molecules: machine learning aided first principles calculations’ by Ryosuke Jinnouchi *et al.*, *Chem. Sci.*, 2025, **16**, 2335–2343, https://doi.org/10.1039/D4SC03378G.

The authors regret that in the original manuscript, a systematic error was present in the calculation of vibrational quantum corrections for the solvated proton.

Specifically, in the classical harmonic oscillator model used to evaluate the nuclear quantum contribution to the free energy *via* eqn (S24), base-10 logarithms were mistakenly used instead of natural logarithms. The corrected version of eqn (S24) is shown below.*A*_c,vib_ = −*k*_B_*T* ln(*hν*_i_/*k*_B_*T*) (S24)

This error resulted in an overestimation of the free energy of the solvated proton by approximately 0.1 eV, which in turn caused an upward shift in both the real potential and the absolute standard hydrogen electrode potential (ASHEP), as presented in Table 1.

Although this correction also leads to minor changes in the plot of the redox potential for the 2H^+^/H_2_ couple and the RMSE bar in Fig. 4, the visual differences are subtle and not easily discernible. The main conclusion of the study remains unchanged.

The corrected values for the real potential, ASHEP, and the vibrational quantum corrections are provided in the revised versions of Tables 1 and S5 shown below.

**Table 1:** Real potential of proton 
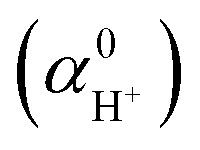
 (eV), ASHEP (V) and relevant free energies (eV) calculated by five exchange–correlation functionals (RPBE+D3, PBE0, PBE0+D3, HSE06 and B3LYP) compared with the experimental values recommended by the International Union of Pure and Applied Chemistry (IUPAC).^1^ Δ_at_*E* and Δ_at_*G*^0^ represent the atomization energy and dissociation free energy of the H_2_ molecule, respectively. Δ_ion_*G*^0^ is the ionization potential of an H atom in vacuum. MLFF denotes the machine-learned force field trained on the RPBE+D3 data. The specified modelling error bars correspond to 2*σ*, estimated by block averaging analysis.^2^ The corrected values are highlighted in bold for clarity

	Δ_at_*E*	Δ_at_*G*^0^	Δ_ion_*G*^0^	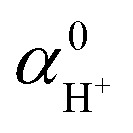	ASHEP
MLFF	4.58	4.04	13.75	**−11.09** ± 0.05	−**4.68** ± 0.05
RPBE+D3	4.58	4.04	13.75	**−11.12** ± 0.06	−**4.65** ± 0.05
PBE0	4.53	3.99	13.64	**−11.15** ± 0.09	−**4.48** ± 0.09
PBE0+D3	4.53	3.99	13.64	−**11.21** ± 0.09	−**4.42** ± 0.09
HSE06	4.53	3.99	13.63	−**11.15** ± 0.09	−**4.47** ± 0.09
B3LYP	4.78	4.25	13.67	−**11.02** ± 0.08	−**4.77** ± 0.09
Exp.	4.73	4.21	13.62	−11.28 ± 0.02	−4.44 ± 0.02

**Table S5** Nuclear quantum effects on the free energies of H_2_O and H_3_O^+^ isolated in vacuum estimated as the difference between the quantum oscillator model and the harmonic oscillator model. The estimation using the experimental vibrational frequencies of solvated proton is also listed. Units of the free energy and vibrational frequencies are eV and cm^−1^, respectively. The corrected values are highlighted in bold for clarity.

**Table:** 

Species	Property	RPBE+D3	PBE0	PBE0+D3	Exp.
H_2_O	*ν*_i_	3831	4020	4020	
		3702	3886	3885	
		1592	1611	1611	
	*A*_q,vib_	0.566	0.590	0.590	
	*A*_c,vib_	**0.201**	**0.204**	**0.204**	
	*A*_q−c_	**0.365**	**0.386**	**0.386**	
	ZPE	0.566	0.590	0.590	
H_3_O^+^	*ν*_i_	3599	3761	3761	3020
		3598	3760	3760	
		3482	3650	3649	
		1647	1669	1669	1760
		1638	1652	1651	1250
		802	688	688	
	*A*_q,vib_	0.916	0.941	0.941	0.374
	*A*_c,vib_	**0.360**	**0.361**	**0.360**	**0.170**
	*A*_q−c_	**0.556**	**0.580**	**0.580**	**0.204**
	ZPE	0.916	0.942	0.942	0.374
	Correction to 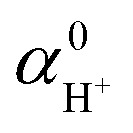	**0.190**	**0.194**	**0.194**	**0.204**
	ZPE[H_3_O^+^]–ZPE[H_2_O]	0.350	0.351	0.351	0.374

The Royal Society of Chemistry apologises for these errors and any consequent inconvenience to authors and readers.
