# Supplementary material for: Absolute standard hydrogen electrode potential and redox potentials of atoms and molecules: machine learning aided first principles calculations
Source: Chem Sci. 2024 Dec 23;16(5):2335–43. doi: 10.1039/d4sc03378g (PMC11702039; doi:10.1039/d4sc03378g)
Supplement: SC-016-D4SC03378G-s001 [file SC-016-D4SC03378G-s001.pdf]

Supplementary Information (SI) for Chemical Science.
This journal is © The Royal Society of Chemistry 2025

Electronic Supplementary Information for Absolute standard hydrogen electrode potential and redox potentials of atoms and molecules: machine learning aided first principles calculations

Ryosuke Jinnouchi

Toyota Central R&D Labs., Inc., Yokomichi 41-1, Nagakute, Aichi, Japan. *

Ferenc Karsai

VASP Software GmbH, Berggasse 21, A-1090 Vienna, Austria.

Georg Kresse†

University of Vienna, Faculty of Physics,

Kolingasse 14-16, A-1090 Vienna, Austria.

* jryosuke@mosk.tytlabs.co.jp

† Also at VASP Software GmbH.

S1. MLFF AND Δ-ML

Similar to previous ML approaches [1, 2], in our MLFF method [3], the potential energy U of a structure with N_a atoms is approximated as a summation of local energies U_i, as written below:

$$U = \sum_{i=1}^{N_\mathrm{a}} U_i. \tag{S1}$$

Following the Gaussian approximation potential pioneered by Bártok and collaborators, the local energy U_i is approximated as a weighted sum of functions $K\left(\mathbf{x}_i, \mathbf{x}_{i_\mathrm{B}}\right)$ centered at reference points $\{\mathbf{x}_{i_\mathrm{B}} | i_\mathrm{B} = 1, ..., N_\mathrm{B}\}$:

$$U_i = \sum_{i_\mathrm{B}=1}^{N_\mathrm{B}} w_{i_\mathrm{B}} K\left(\mathbf{x}_i, \mathbf{x}_{i_\mathrm{B}}\right). \tag{S2}$$

Here, the descriptors of fingerprints $\mathbf{x}_i = \mathbf{x}_i\left[\rho_i\left(\mathbf{r}\right)\right]$ are rotationally and translationally invariant functionals of the density distribution around atom i [3–5],

$$\rho_i\left(\mathbf{r}\right) = \sum_{j=1}^{N_\mathrm{a}} f_\mathrm{cut}\left(\left|\mathbf{r}_j - \mathbf{r}_i\right|\right) g\left(\mathbf{r} - \left(\mathbf{r}_j - \mathbf{r}_i\right)\right), \tag{S3}$$

where the function g is a smoothed δ-function, and f_cut is a cutoff function that smoothly eliminates the contribution from atoms outside a given cutoff radius R_cut. The function $K\left(\mathbf{x}_i, \mathbf{x}_{i_\mathrm{B}}\right)$ measures the similarity in the local structure surrounding atom i and atom i_B in the reference structures. The reference structures are selected from the training set. For the kernel basis functions, the smooth overlap of atomic positions (SOAP) kernel [4] is employed.

$$K\left(\mathbf{x}_i, \mathbf{x}_{i_\mathrm{B}}\right) = \left(\hat{\mathbf{x}}_i \cdot \hat{\mathbf{x}}_{i_\mathrm{B}}\right)^\zeta. \tag{S4}$$

The hat symbol $\hat{\mathbf{x}}_i$ denotes a normalized vector of $\mathbf{x}_i$. The normalization and exponentiation in Eq. (S4) introduce non-linear terms that mix two- and three-body contributions. The coefficients $\{w_{i_\mathrm{B}} | i_\mathrm{B} = 1, ..., N_\mathrm{B}\}$ are optimized to best reproduce the FP energies, forces, and stress tensor components as obtained by the FPMD simulations.

The same formulation is used for the Δ-ML method. In the Δ-ML method, differences of potential energies and forces between two FP methods, semi-local and non-local functionals in

this study, are used as the training data.

Parameter sets of the descriptors and kernel basis functions used in previous publications [3, 5, 6] were employed in this study. The parameters are tabulated in Table S1. See details of the tabulated symbols in the previous publications.

The unit cells used for training MLFFs are shown in Fig. S 1. The MLFF was trained on systems consisting of 64 H_2O molecules and H^++64H_2O molecules inside cubes with a side length of 12.4 Å. The selection of the 64 water molecule system was based on a previous study that demonstrated sufficient convergence with this system size. The MLFFs for V^{3+}/V^{2+} and Ru^{3+}/Ru^{2+} were trained on systems consisting of X+64H_2O molecules, where X denotes the redox species. The MLFF for O_2/O_2^- redox couples was trained on O_2/O_2^-+62H_2O molecular systems. The MLFFs for Fe^{3+}/Fe^{2+}, Cu^{2+}/Cu^+, and Ag^{2+}/Ag^+ are the same as those in our previous publication [7]. The MLFF for H^++64H_2O was generated on the fly during a 200 ps NVT-ensemble MD simulation ramping up the temperature from 300 K to 500 K for each of the 64 H_2O and H^++64H_2O systems. Prior to the production runs, in addition to the heating simulation, the MLFF was trained on the fly during the TI simulation from the repulsive surrogate model to the fully interacting system as explained in Section S2. This additional training is similar to the method proposed by Wang and co-workers [8, 9]. In our on-the-fly active learning scheme, as in the production run for TI explained in Section S2, MD simulations are performed on potential energy surfaces that are linear combinations of the MLFFs for the reactant and product states. At every MD step, uncertainties in the predictions are evaluated using the spilling factor. [3] FP calculations are carried out only for structures with high uncertainties, and the new FP data obtained in this way are then used to update the MLFFs. Similar to the conventional on-the-fly training method [3], by restricting FP calculations to only those structures with high uncertainties, we can significantly reduce the number of required FP calculations. This additional training allows for the collection of configurations that appear in the TI along the thermodynamic path of proton insertion, significantly stabilizing the TI simulations in the production runs. The MLFFs for V^{3+}/V^{2+}, Ru^{3+}/Ru^{2+}, and O_2/O_2^-+62H_2O were generated by the same method. In all MD simulations, the temperature was controlled using the Langevin thermostat [10]. For an efficient sampling of training configurations, the mass of hydrogen atoms was increased to 4.0 a.u., and the time-step was set to 2.0 fs in all training MD simulations.

For each of the reactant and product states, a Δ-ML model was generated. For each state, a 100 ps NVT-ensemble MD simulation was conducted using the MLFF trained on RPBE+D3,

and 40 configurations were randomly selected from the MD trajectory. FP calculations were then performed on these configurations using the PBE0, PBE0+D3, HSE06, and B3LYP functionals. The differences in energies and forces between the semi-local functional (RPBE+D3) and each of the other functionals were used to train the Δ-ML model.

In all FP calculations, the plane-wave cutoff energy was set to 520 eV. The Γ-point was used for Brillouin zone integration. The PAW atomic reference configurations were $1s^1$ for H, $2s^2 2p^4$ for O, $3s^2 3p^6 3d^4 4s^1$ for V, $3d^7 4s^1$ for Fe, $3d^{10} 4s^1$ for Cu, $4s^2 4p^6 4d^7 5s^1$ for Ru, and $4d^{10} 5s^1$ for Ag.

The number of structures, N_{st}, for each system is provided in Table S2. The training datasets can be accessed from the repository [11]. The root mean square errors (RMSEs) of energies and forces predicted by the MLFF and Δ-ML models are tabulated in Tables S3 and S4, respectively. The probability densities for finding specific differences in potential energy and force between the ML and FP results are summarized in Figures S2 to S8.

Table S 1. Parameter sets of descriptors and kernel basis functions.

Parameters for H^++64H_2O											
ζ	4	$\beta^{(2)}$	0.5	$R_{\mathrm{cut}}^{(2)}$	6.0	$\sigma_{\mathrm{atom}}^{(2)}$	0.5	$N_{\mathrm{R}}^{(2)}$	8		
		$\beta^{(3)}$	0.5	$R_{\mathrm{cut}}^{(3)}$	4.0	$\sigma_{\mathrm{atom}}^{(3)}$	0.5	$N_{\mathrm{R}}^{(3)}$	6	$L_{\mathrm{max}}^{(3)}$	3
Parameters for other systems											
ζ	4	$\beta^{(3)}$	1.0	$R_{\mathrm{cut}}^{(3)}$	5.0	$\sigma_{\mathrm{atom}}^{(3)}$	0.5	$N_{\mathrm{R}}^{(3)}$	8	$L_{\mathrm{max}}^{(3)}$	4

Table S 2. The numbers of structures N_{st} calculated by the FP method to generate training data for the MLFFs.

System	N_{st}	System	N_{st}	System	N_{st}	System	N_{st}
64H_2O	189	H^++64H_2O	399	V^{3+}+64H_2O	89	V^{2+}+64H_2O	77
Fe^{3+}+64H_2O	162	Fe^{2+}+64H_2O	97	Cu^{2+}+64H_2O	173	Cu^++64H_2O	266
Ru^{3+}+64H_2O	149	Ru^{2+}+64H_2O	144	Ag^{2+}+64H_2O	73	Ag^++64H_2O	181
O_2+62H_2O	407	O_2^-+62H_2O	319				

Table S 3. Root mean square errors (RMSE) for MLFF in energy σ_E (meV atom^{-1}), forces σ_F (eV Å^{-1}), and stress tensor components σ_S (kbar). The RMSE was evaluated for each system using 100 test structures taken from a 10 ps TI simulation from MLFF to RPBE+D3.

System	σ_E	σ_F	σ_S
$64H_2O$	1.1	0.04	0.85
H^++$64H_2O$	0.6	0.05	0.59
V^{3+}+$64H_2O$	1.4	0.07	0.51
V^{2+}+$64H_2O$	1.4	0.06	0.47
Fe^{3+}+$64H_2O$	1.3	0.07	0.62
Fe^{2+}+$64H_2O$	1.2	0.07	0.59
Cu^{2+}+$64H_2O$	1.1	0.07	1.07
Cu^++$64H_2O$	0.9	0.06	0.78
Ru^{3+}+$64H_2O$	1.8	0.08	0.84
Ru^{2+}+$64H_2O$	2.2	0.07	0.71
Ag^{2+}+$64H_2O$	1.6	0.07	1.08
Ag^++$64H_2O$	1.2	0.05	0.84
O_2+$62H_2O$	2.0	0.05	0.44
O_2^-+$62H_2O$	0.9	0.05	0.44

Table S 4. Root mean square errors (RMSEs) of Δ-MLs for differences of energies σ_E (meV atom^{-1}) and forces σ_F (eV Å^{-1}) between the semi-local (RPBE+D3) and hybrid functional (PBE0 or PBE0+D3) compared to FP test data. Here, RMSE is evaluated for each system using 50 test structures taken from a 100 ps MD trajectory of RPBE+D3.

	PBE0 - RPBE+D3		PBE0+D3 - RPBE+D3	
System	σ_E	σ_F	σ_E	σ_F
$64H_2O$	0.05	0.004	0.05	0.004
H^++$64H_2O$	0.05	0.005	0.05	0.005
V^{3+}+$64H_2O$	0.10	0.008	0.11	0.008
V^{2+}+$64H_2O$	0.06	0.005	0.06	0.006
Fe^{3+}+$64H_2O$	0.05	0.006	0.05	0.006
Fe^{2+}+$64H_2O$	0.06	0.006	0.06	0.006
Cu^{2+}+$64H_2O$	0.06	0.007	0.07	0.007
Cu^++$64H_2O$	0.04	0.005	0.04	0.005
Ru^{3+}+$64H_2O$	0.24	0.010	0.24	0.009
Ru^{2+}+$64H_2O$	0.05	0.005	0.05	0.006
Ag^{2+}+$64H_2O$	0.07	0.007	0.07	0.007
Ag^++$64H_2O$	0.04	0.005	0.01	0.004
O_2+$62H_2O$	0.16	0.009	0.17	0.009
O_2^-+$62H_2O$	0.07	0.007	0.07	0.007

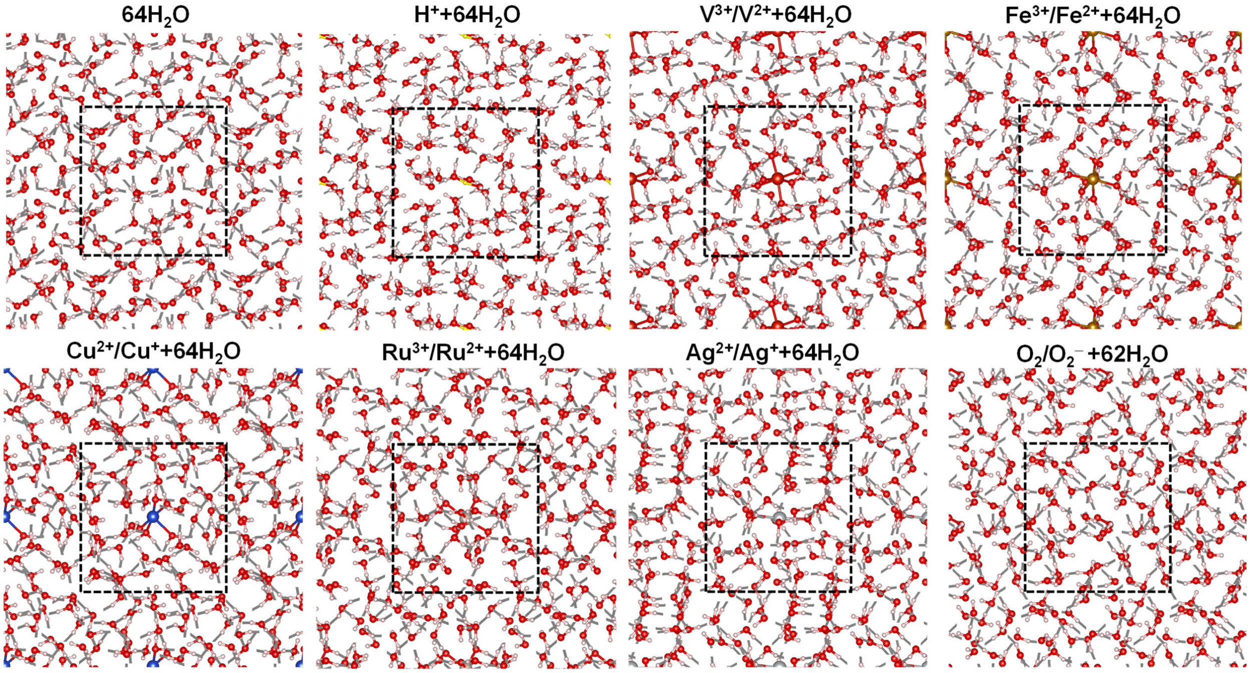

Figure S 1. Models used for training and production runs. Dashed squares represent the unit cells.

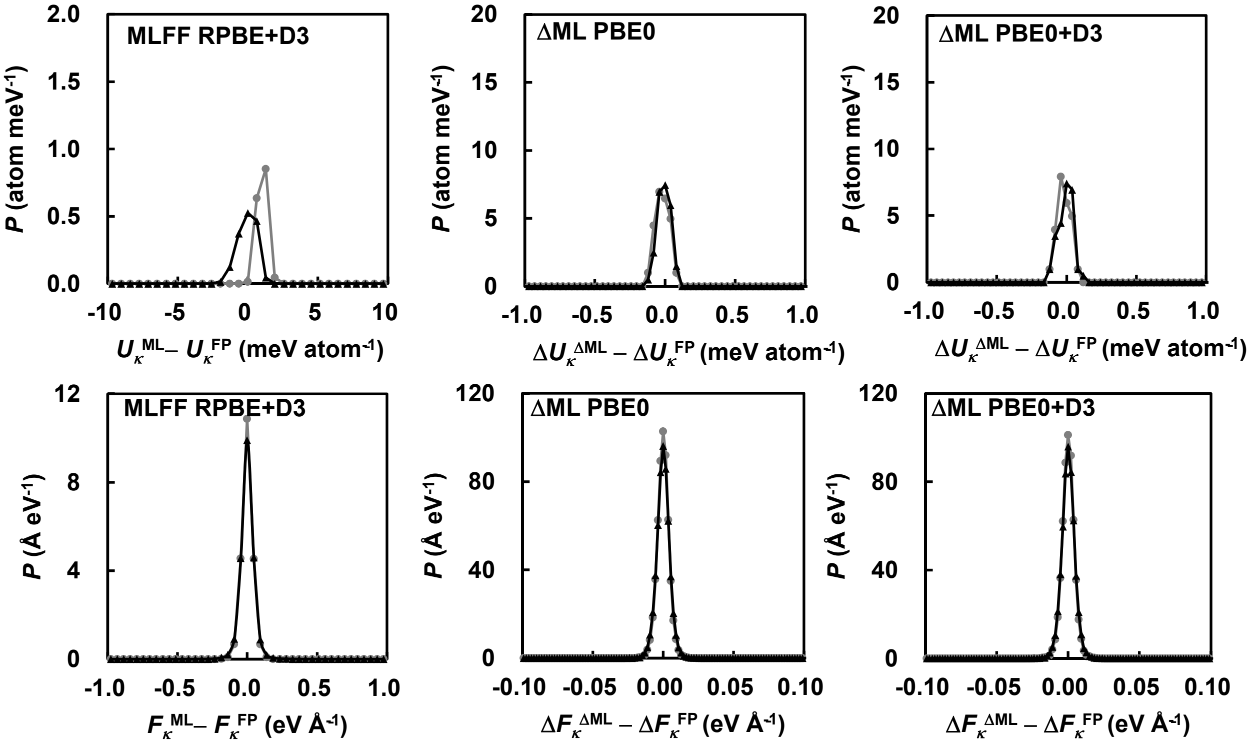

Figure S 2. Probability densities P to find specific differences between the ML and FP results for the $64H_2O$ (gray) and H^++$64H_2O$ (black) bulk solutions. U_κ and F_κ denote the potential energy and force, respectively, at the state κ (κ=0 for $64H_2O$ and $\kappa = 1$ for H^++$64H_2O$). ΔU_κ and ΔF_κ denote the differences of the potential energy and force, respectively, between the hybrid functional [(PBE0 and PBE0+D3] and the semi-local functional (RPBE+D3). The superscript 'ΔML' and 'FP' indicate the values calculated by the Δ-ML models and the FP method.

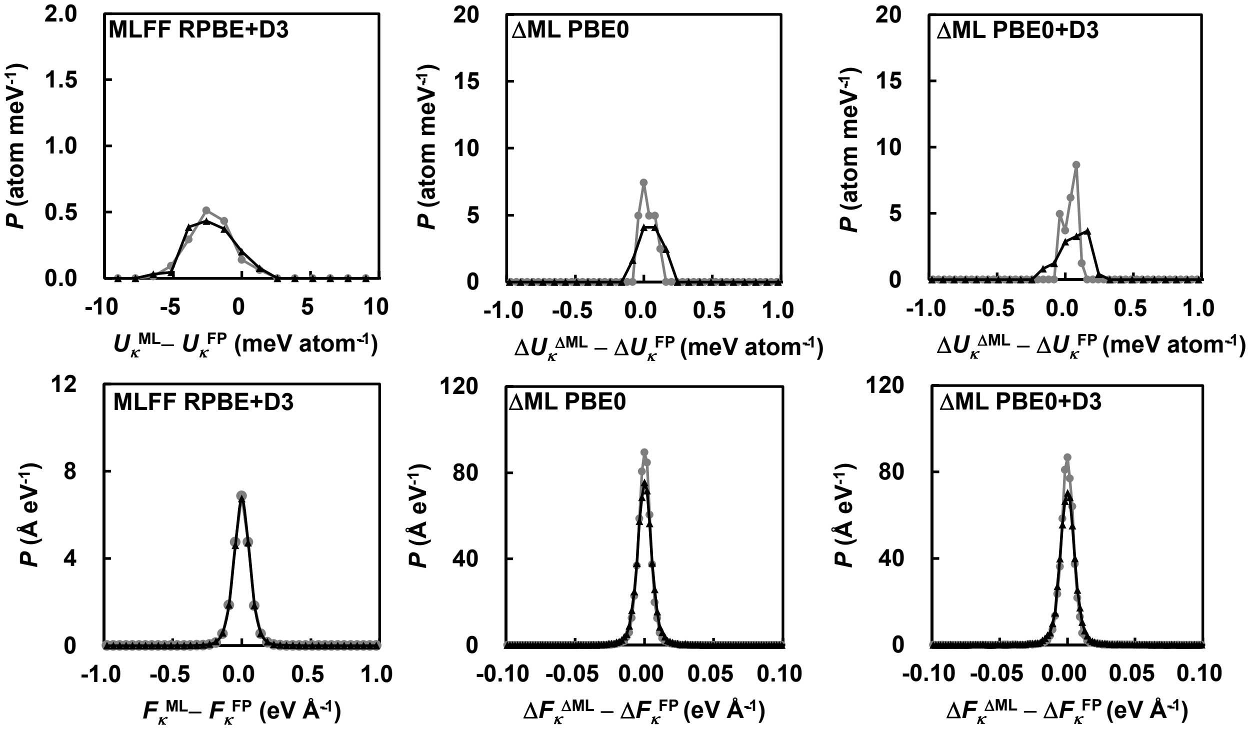

Figure S 3. Probability densities P to find specific differences between the ML and FP results for the V^{3+}+64H_2O (black) and V^{2+}+64H_2O (gray) bulk solutions. Here, κ=0 denotes the oxidized state, and κ=1 denotes the reduced state. Other notations are the same as the ones in Fig. S2.

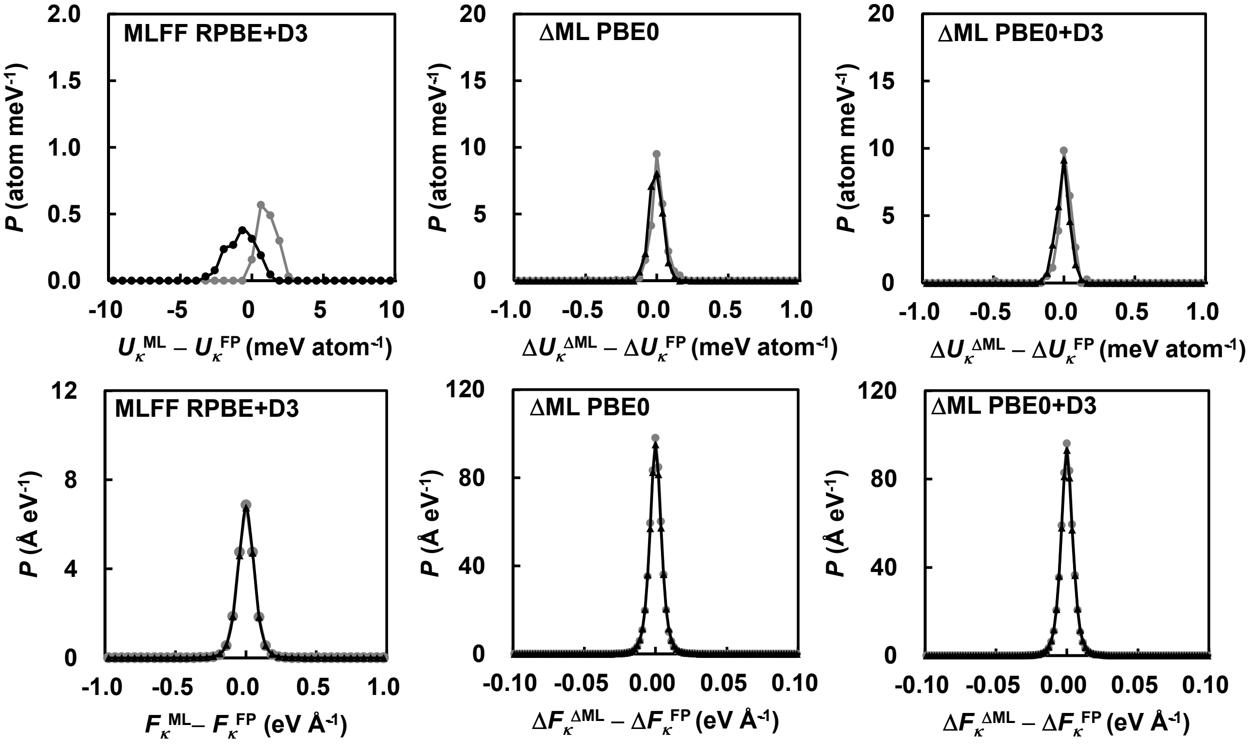

Figure S 4. Probability densities P to find specific differences between the ML and FP results for the Fe^{3+}+64H_2O (black) and Fe^{2+}+64H_2O (gray) bulk solutions. Notations are the same as the ones in Fig. S3.

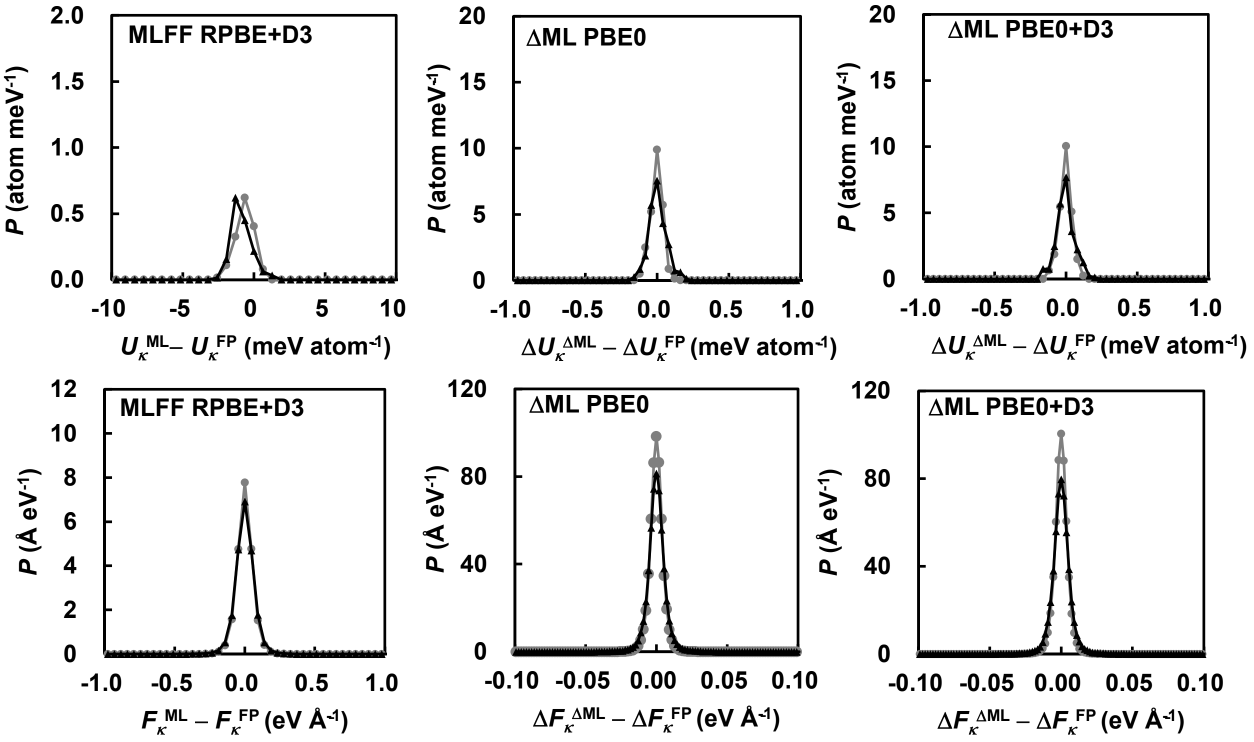

Figure S 5. Probability densities P to find specific differences between the ML and FP results for the Cu^{2+}+64H_2O (black) and Cu^{+}+64H_2O (gray) bulk solutions. Notations are the same as the ones in Fig. S3.

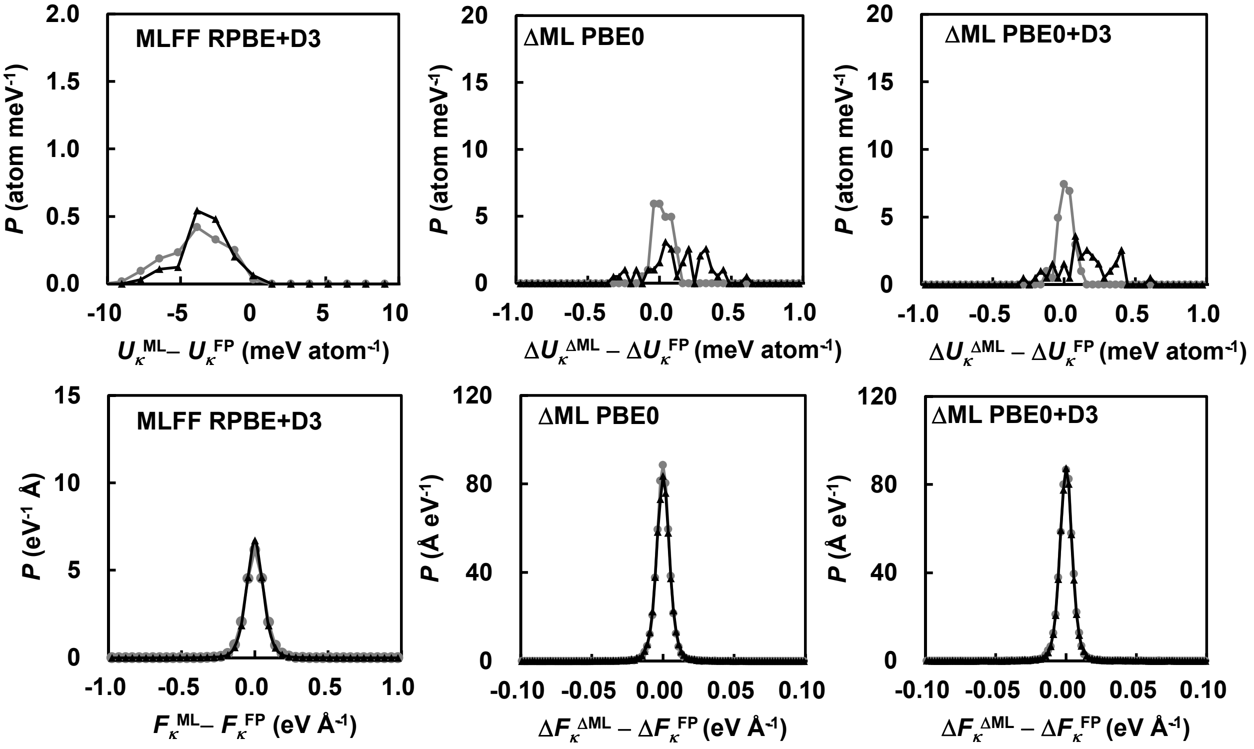

Figure S 6. Probability densities P to find specific differences between the ML and FP results for the Ru^{3+}+64H_2O (black) and Ru^{2+}+64H_2O (gray) bulk solutions. Notations are the same as the ones in Fig. S3.

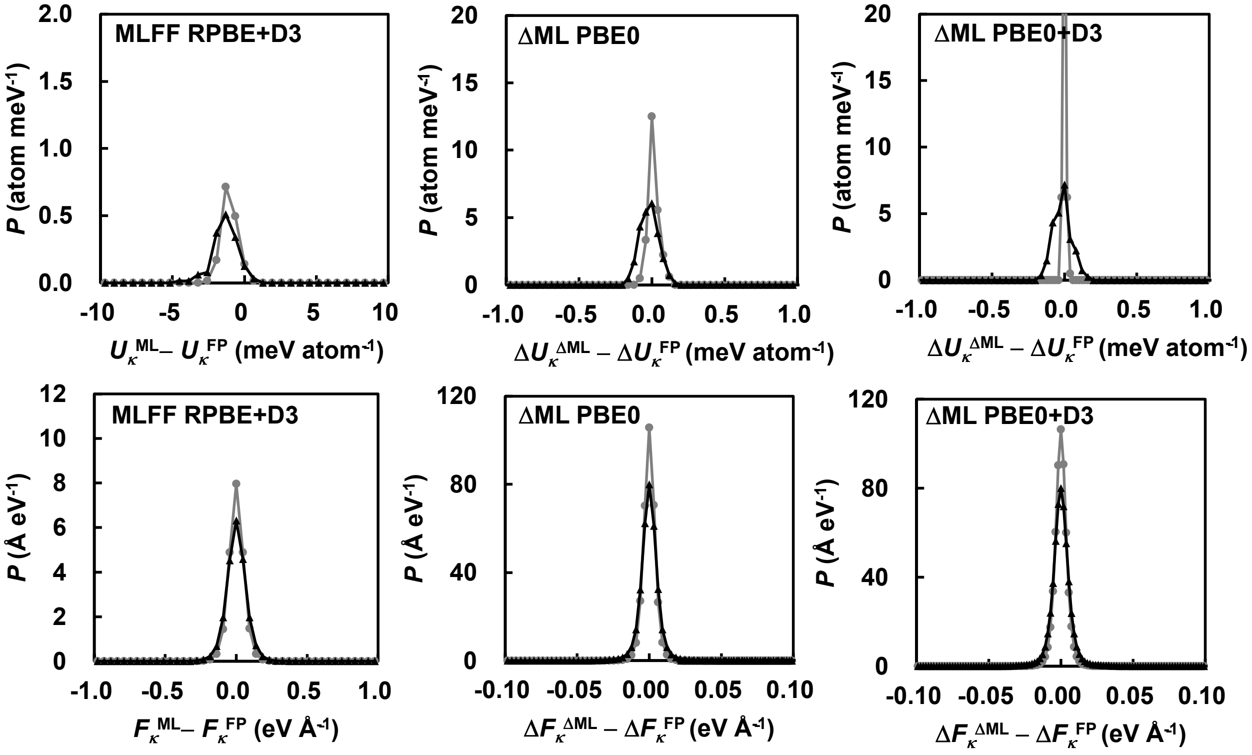

Figure S 7. Probability densities P to find specific differences between the ML and FP results for the Ag^{2+}+64H_2O (black) and Ag^{+}+64H_2O (gray) bulk solutions. Notations are the same as the ones in Fig. S3.

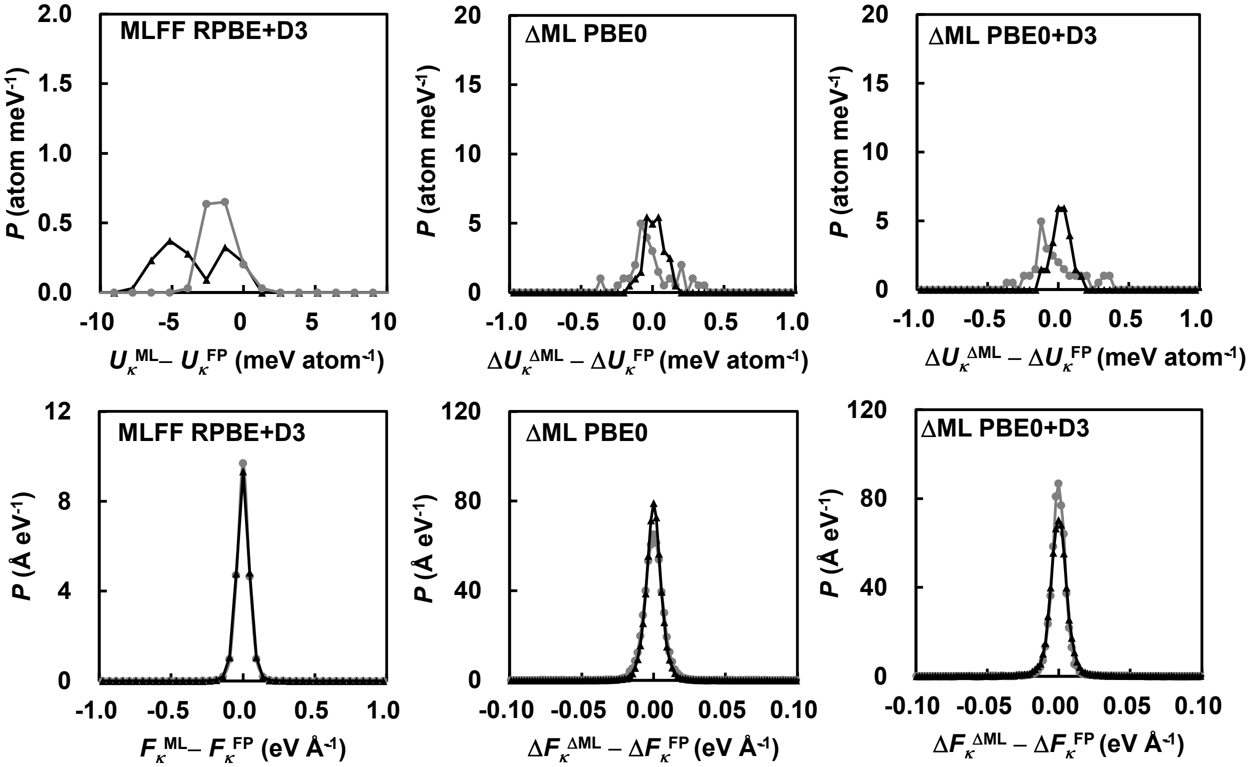

Figure S 8. Probability densities P to find specific differences between the ML and FP results for the O_2+64H_2O (black) and O_2^-+64H_2O (gray) bulk solutions. Notations are the same as the ones in Fig. S3.

S2. TI AND TPT

The TI and TPT simulations used for the electron transfer reactions of the three redox couples, V^{3+}/V^{2+}, Ru^{3+}/Ru^{2+}, and O_2/O_2^-, are the same as those employed in our previous publication [7]. In these methods, MLFF surrogate models, which enable the necessary electron insertions into the aqueous systems, are utilized to efficiently perform statistical sampling. The results for the electron transfer reactions of six redox couples, V^{3+}/V^{2+}, Fe^{3+}/Fe^{2+}, Cu^{2+}/Cu^{+}, Ru^{3+}/Ru^{2+}, and Ag^{2+}/Ag^{+} and O_2/O_2^-, are summarized in Fig. S 9.

In this study, the method was modified to extend it to proton insertion to compute the real potential of the proton. Here, we consider the forward process of proton solvation [$H^+(g) \rightarrow H^+(aq)$]. The symbols (g) and (aq) denote the species in the gas phase and aqueous phase, respectively. The equations are summarized below.

- $\mathbf{ML}(\mathbf{R}) \rightarrow \mathbf{ML}(\mathbf{P})$

$$\Delta A^{\mathrm{ML}} = \int_0^1 \left\langle \frac{\partial H^{\mathrm{ML}}}{\partial \lambda} \right\rangle_{\lambda} d\lambda, \tag{S5}$$

$$H^{\mathrm{ML}} = \sum_{i=1}^{N_{\mathrm{a}}} \frac{|\mathbf{p}_i|^2}{2m_i} + \lambda \left(U_1^{\mathrm{ML}} + e\Delta\phi \right) + (1-\lambda)\, U_0^{\mathrm{ML}}. \tag{S6}$$

- $\mathbf{ML}(\mathbf{R}) \rightarrow \mathbf{FP_{sl}}(\mathbf{R})$ and $\mathbf{ML}(\mathbf{P}) \rightarrow \mathbf{FP_{sl}}(\mathbf{P})$

$$\Delta A_{\kappa}^{\mathrm{FP_{sl}-ML}} = \int_0^1 \left\langle \frac{\partial H_{\kappa}^{\mathrm{FP_{sl}-ML}}}{\partial \eta} \right\rangle_{\eta} d\eta, \tag{S7}$$

$$H_{\kappa}^{\mathrm{FP_{sl}-ML}} = \sum_{i=1}^{N_{\mathrm{a}}} \frac{|\mathbf{p}_i|^2}{2m_i} + \eta U_{\kappa}^{\mathrm{FP_{sl}}} + (1-\eta)\, U_{\kappa}^{\mathrm{ML}}. \tag{S8}$$

- $\mathbf{FP_{sl}}(\mathbf{R}) \rightarrow \mathbf{FP_{nl}}(\mathbf{R})$ and $\mathbf{FP_{sl}}(\mathbf{P}) \rightarrow \mathbf{FP_{nl}}(\mathbf{P})$

$$\Delta A_{\kappa}^{\mathrm{FP_{nl}-FP_{sl}}} \simeq \left\langle \Delta U_{\kappa}^{\Delta\mathrm{ML}} \right\rangle_{\mathrm{FP_{sl}}} - \frac{\beta}{2} \left\langle \left(\Delta U_{\kappa}^{\Delta\mathrm{ML}} - \left\langle \Delta U_{\kappa}^{\Delta\mathrm{ML}} \right\rangle_{\mathrm{FP_{sl}}} \right)^2 \right\rangle_{\mathrm{FP_{sl}}}. \tag{S9}$$

The symbols R and P denote the reactant state and the product state, respectively. $\mathrm{FP_{nl}}$(R/P), $\mathrm{FP_{sl}}$(R/P), and ML(R/P) denote calculations using a non-local hybrid functional, a semi-local functional and machine-learned force field for the reactant and product states, respectively. The symbol

N_a represents the number of atoms, and m_i and $\mathbf{p}_i$ represent the mass and momentum vector of the i-th atom, respectively. The symbols $U_\kappa^{\mathrm{FP_{nl}}}$, $U_\kappa^{\mathrm{FP_{sl}}}$, and U_κ^{ML} denote the potential energies for the reactant state ($\kappa = 0$) and the product state ($\kappa = 1$) calculated by the non-local functional, semi-local functional, and MLFF trained on the semi-local functional, respectively. The symbol $\Delta U_\kappa^{\Delta\mathrm{ML}}$ denotes the potential energy difference calculated by the Δ-ML model trained on the potential energy difference $U_\kappa^{\mathrm{FP_{nl}}} - U_\kappa^{\mathrm{FP_{sl}}}$ between the non-local and semi-local functionals. Equation (S9) uses the second-order cumulant expansion. The expansion is exact if the probability distribution of $\Delta U_\kappa^{\Delta\mathrm{ML}}$ is Gaussian. This condition is reasonably satisfied, as shown in Fig. 2 (e) in the main text. These equations are the same as those for the electron insertion method developed in our previous study [7]. The difference lies only in the representation of the reactant state (R) and the product state (P). In the previous electron insertion method, R and P were the respective states before and after the electron insertion. In the proton insertion method, they represent the states before and after the proton is inserted into the aqueous medium. Although the difference is only in the species being inserted, in the case of the proton, the structure of water changes significantly before and after the insertion, making it very difficult to generate a stable and reversible path of proton insertion in the TI Eq. (S5). To address this issue, we developed the soft-landing scheme. Details of the TI scheme and the TPT simulation are explained below. The results of the TI and TPT simulations for H^+ solvation are shown in Fig. 2 in the main text.

TI simulation using MLFF: In the TI equation (S5), a single proton is gradually inserted into the interacting system by gradually turning on the MLFF interaction of the proton as the coupling parameter λ increases. At the initial stage, the not-yet-interacting proton can come close to another atom. If the interaction is calculated by the FP method, the atoms should experience a large repulsive potential. However, an MLFF not trained for such unrealistic structures may erroneously provide lower energies. If this occurs in the TI process, the system is irreversibly trapped in this false virtual well, leading to incorrect free energy in the simulation. To avoid this problem, we introduce a model potential that supports a soft landing on the realistic structure of the interacting system, as done in our previous publication [12]. The soft-landing scheme consists of the following

two stages of TI simulations:

$$\Delta A^{\mathrm{ML}} = \Delta A^{\mathrm{ML}}_{\mathrm{I}} + \Delta A^{\mathrm{ML}}_{\mathrm{II}}, \tag{S10}$$

$$\Delta A^{\mathrm{ML}}_{\mathrm{I}} = \int_0^1 \left\langle \frac{\partial H^{\mathrm{ML}}_{\mathrm{I}}}{\partial \lambda_{\mathrm{I}}} \right\rangle_{\lambda_{\mathrm{I}}} d\lambda_{\mathrm{I}}, \tag{S11}$$

$$H^{\mathrm{ML}}_{\mathrm{I}} = \sum_{i=1}^{N_{\mathrm{a}}} \frac{|\mathbf{p}_i|^2}{2m_i} + \lambda_{\mathrm{I}} U_{\mathrm{model}} + \sum_{i \notin \mathrm{M}} U_i(0), \tag{S12}$$

$$\Delta A^{\mathrm{ML}}_{\mathrm{II}} = \int_0^1 \left\langle \frac{\partial H^{\mathrm{ML}}_{\mathrm{II}}}{\partial \lambda_{\mathrm{II}}} \right\rangle_{\lambda_{\mathrm{II}}} + e\int_0^1 \langle \Delta\phi \rangle_{\lambda_{\mathrm{II}}} d\lambda_{\mathrm{II}}, \tag{S13}$$

$$H^{\mathrm{ML}}_{\mathrm{II}} = \sum_{i=1}^{N_{\mathrm{a}}} \frac{|\mathbf{p}_i|^2}{2m_i} + \lambda_{\mathrm{II}} \sum_{i=1}^{N_{\mathrm{a}}} U_i(1) + (1-\lambda_{\mathrm{II}}) \left[U_{\mathrm{model}} + \sum_{i \notin \mathrm{M}} U_i(0) \right]. \tag{S14}$$

Here, the symbol M denotes the inserting species, which is a single proton in this study. As explained in the previous subsection regarding the kernel-based approach, the atomic potential energies [$U_i(0)$ and $U_i(1)$] are expressed as a linear combination of functions $K(\mathbf{x}_i(\lambda), \mathbf{x}_{i_{\mathrm{B}}})$, similar to what was described before:

$$U_i(\lambda) = \sum_{i_{\mathrm{B}}=1}^{N_{\mathrm{B}}} w_{i_{\mathrm{B}}} K(\mathbf{x}_i(\lambda), \mathbf{x}_{i_{\mathrm{B}}}). \tag{S15}$$

Here, the descriptors $\mathbf{x}_i(\lambda) = \mathbf{x}_i[\rho_i(\mathbf{r},\lambda)]$ are the functional of the density distribution function around atom i,

$$\rho_i(\mathbf{r},\lambda) = \sum_{j \notin \mathrm{M}} f_{\mathrm{cut}}(|\mathbf{r}_j - \mathbf{r}_i|)\, g(\mathbf{r} - (\mathbf{r}_j - \mathbf{r}_i)) + \lambda \sum_{j \in \mathrm{M}} f_{\mathrm{cut}}(|\mathbf{r}_j - \mathbf{r}_i|)\, g(\mathbf{r} - (\mathbf{r}_j - \mathbf{r}_i)). \tag{S16}$$

Hence, $U_i(0)$ and $U_i(1)$ give the local energies without and with the group M. Using these notations, $U^{\mathrm{ML}}_{\mathrm{R}}$ and $U^{\mathrm{ML}}_{\mathrm{P}}$ can be written as $\sum_{i \notin \mathrm{M}} U_i(0)$ and $\sum_{i=1}^{N_{\mathrm{a}}} U_i(1)$. It should be noted that the potential energy of the product state in Eq. (S15) is shifted by $e\Delta\phi$ as explained in Section 2 in the main text. This system-dependent shift is corrected by the second term on the right-hand side of Eq. (S13). Details of how to compute $\Delta\phi$ are described in Section S4.

The additional model potential U_{model} is designed to avoid erroneous predictions on unrealistic structures by the MLFF $U_i(1)$. In the first TI simulation equation (S11), unrealistic structures appear near the non-interacting limit (λ_{I}=0 and λ_{II}=0). However, because the MLFF $U_i(0)$ ignores the inserting proton, it can accurately predict the potential energy of water molecules. Conse-

quently, the TI stably generates a cavity in water. In the second TI simulation equation (S13), because the MLFF $U_i(1)$ has already learned all possible configurations of the fully interacting solution involving proton, with and without the cavity, it can accurately predict the potential energy of the solution. The soft repulsive potential U_{model} is modeled by a simple Gaussian potential as follows:

$$U_{\text{model}} = \sum_{i \in \text{M}} \sum_{j \notin \text{M}} a_0 e^{-\left(\frac{r_{ij}}{r_0}\right)^2}. \tag{S17}$$

The summation over i is taken over all atoms in the group M, and the summation over j is taken over all atoms except those in the group M. The maximum height of the repulsive potential a_0 was set to 10 eV, and the radius r_0 was set as follows:

$$r_0 = \frac{l_{\text{vdw}}}{\sqrt{-\ln\left(k_{\text{B}}T/a_0\right)}}. \tag{S18}$$

The repulsive length l_{vdw} was set to the sum of the van der Waals radii of H and O atoms. However, it should be noted that the final real potential of the proton is insensitive to these parameters if sufficient statistical sampling is conducted.

The integration over the coupling parameter λ_{I} in Eq. (S11) and the one over the λ_{II} in the first term on the right-hand side of Eq. (S13) were performed using numerical integration. As shown in Fig. 2(b) in the main text, the integrands undergo rapid changes near $\lambda_{\text{I}} = 0$. Near $\lambda_{\text{I}} = 0$, the interaction between the inserted atom and other atoms becomes infinitesimally small, allowing the yet uninteracting atom to come very close to the other atom and experience the repulsive Gaussian potential. To achieve accurate numerical integration, the following variable transformations were employed for both λ_{I} and λ_{II} [13]:

$$\lambda = \left(\frac{x+1}{2}\right)^{\frac{1}{1-k}}. \tag{S19}$$

where k is set to 0.5. As in previous studies, after checking for convergence, numerical integration was performed using the 12- and 14-point Gauss-Lobatto quadrature method for the transformed variables x_{I} and x_{II}, respectively [12–14]. To examine reversibility, TI simulations were conducted in two directions. In the first TI, the calculation starts from the origin of the coupling parameter [λ_{I}=0 for Eq. (S11) and λ_{II}=0 for Eq. (S13)]. A 200 ps MD simulation is performed on the

first integration grid, and the final structure from this simulation is used as the initial structure for another 200 ps MD simulation on the next grid. This procedure is repeated until the integration reaches the endpoint [λ_{I}=1 for Eq. (S11) and λ_{II}=1 for Eq. (S13)]. The second thermodynamic integration starts from the endpoint and performs integration towards the origin, following the reverse process of the aforementioned steps. The forward and reverse simulations were repeated 14 times, and by averaging the results of simulations totaling 2.8 ns for each grid, the real potential $\alpha^{0}_{\mathrm{H}^{+}}$ was calculated. The second term on the right-hand side of Eq. (S13) was calculated as the average of $\Delta\phi$ at two endpoints (trapezoidal rule) similarly to our previous study [7].

TI simulation from MLFF to semi-local functional (FP_{sl}): For the TI Eq. (S7), the trapezoidal rule with equidistant three points was used following our previous study. With this setup, the integration error is less than 5 meV. At each point, a 20-ps NVT-ensemble MD simulation at 298 K was performed. The integrands shown in Fig. 2 (d) are smaller than those shown in Fig. 2 (b). They are also nearly proportional to the coupling parameter η. Using the free energy changes $\Delta F_{\kappa}^{\mathrm{FP_{sl}}-\mathrm{ML}}$, the free energy change based on the RPBE+D3 functional is obtained as follows:

$$\Delta A^{\mathrm{FP_{sl}}} = \Delta A^{\mathrm{ML}} + \Delta A_{1}^{\mathrm{FP_{sl}}-\mathrm{ML}} - \Delta A_{0}^{\mathrm{FP_{sl}}-\mathrm{ML}}. \tag{S20}$$

TPT simulation from semi-local functional (FP_{sl}) to non-local hybrid functional (FP_{nl}): In the TPT calculations using the Δ-ML models, the ensemble average for Eq. (S9) was computed over 2000 randomly selected configurations from a trajectory of 100 ps NVT ensemble FPMD simulation using the RPBE+D3 functional. While these FPMD simulations are computationally expensive, the overall computational time is still significantly shorter than that of full FP simulations using the non-local hybrid functional. To ensure the applicability of the second-order cumulant expansion, we show the probability distributions of the energy difference $\Delta U_{\kappa}^{\Delta\mathrm{ML}}$ in Fig. 2 (e). These distributions are well-fitted by Gaussian functions, indicating that Eq. (S9) is a reasonable approximation. The free energy difference based on the non-local hybrid functionals is obtained as follows:

$$\Delta A^{\mathrm{FP_{nl}}} = \Delta A^{\mathrm{FP_{sl}}} + \Delta A_{1}^{\mathrm{FP_{nl}}-\mathrm{FP_{sl}}} - \Delta A_{0}^{\mathrm{FP_{nl}}-\mathrm{FP_{sl}}}. \tag{S21}$$

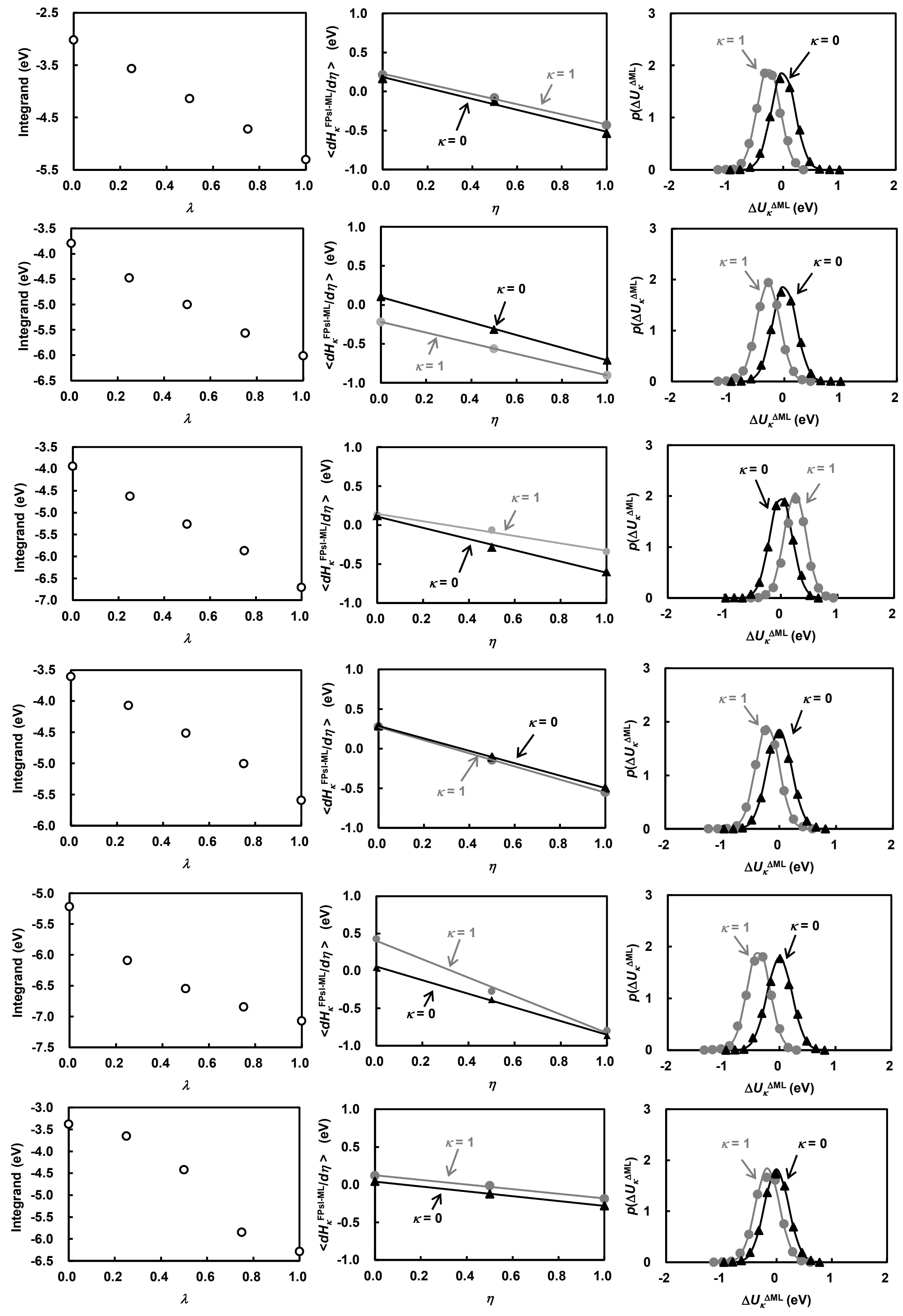

Figure S 9. Summary of the TI and TPT simulations for six redox species, V^{3+}/V^{2+}, Fe^{3+}/Fe^{2+}, Cu^{2+}/Cu^{+}, Ru^{3+}/Ru^{2+}, Ag^{2+}/Ag^{+}, and O_2/O_2^-. The first column shows the integrand of the ML-aided TI equation (S5) from the oxidized state to the reduced state. The second column shows the integrand of Eq. (S7) from the MLFF to RPBE+D3. The third column shows the distribution of the potential energy difference between PBE0+D3 and RPBE+D3 for the structures used for the ensemble averages in Eq. (S9).

S3. CORRECTIONS TO NUCLEAR QUANTUM EFFECTS AND GASEOUS VOLUME

The influence of nuclear quantum effects on the free energy change was estimated using the difference between the quantum oscillator model and the classical oscillator model.

$$\Delta A_{\mathrm{q-c}} = A_{\mathrm{q,vib}} - A_{\mathrm{c,vib}}, \tag{S22}$$

$$A_{\mathrm{q,vib}} = \sum_i \frac{h\nu_i}{2} + \sum_i k_{\mathrm{B}} T \ln\left(1 - e^{-\frac{h\nu_i}{k_{\mathrm{B}}T}}\right), \tag{S23}$$

$$A_{\mathrm{c,vib}} = \sum_i k_{\mathrm{B}} T \ln\left(\frac{h\nu_i}{k_{\mathrm{B}}T}\right), \tag{S24}$$

where h is the Planck constant, and ν_i is the vibrational frequency of the i-th normal mode. Following previous studies [15, 16], we computed the $\Delta A_{\mathrm{q-c}}$ for three vibrational modes observed at 1250, 1760, and 3020 cm^{-1} in experiments [17], assuming that these modes are generated by the solvation of a proton. To examine the sensitivity of the correction to the model and vibrational frequencies used, we also calculated the differences in $\Delta A_{\mathrm{q-c}}$ for H_2O and H_3O^+ isolated in vacuum. Here, the corrections to the free energy change of the proton solvation ($\alpha^0_{\mathrm{H}^+}$) is calculated as $\Delta A_{\mathrm{q-c}}[H_3O^+] - \Delta A_{\mathrm{q-c}}[H_2O]$, where $\Delta A_{\mathrm{q-c}}[*]$ denotes the $\Delta A_{\mathrm{q-c}}$ of the species indicated in the square brackets. Vibrational frequencies were computed by diagonalizing the Hessian matrix for each species placed in a cubic cell with a side length of 20 Å. FP calculations were performed using the parameters explained in Section S1. The obtained vibrational frequencies and free energies are presented in Table S5. In the same table, the zero-point energy contribution, $\mathrm{ZPE}[H_3O^+] - \mathrm{ZPE}[H_2O]$, is also listed for comparison with the corrections of 0.32 to 0.36 eV used in previous studies [15, 16, 18, 19], which only considered the zero-point energy. The examination indicated that the correction is insensitive to the choice of the model and frequcneis. Therefore, we used the experimental vibrational frequencies for the correction.

The difference in free energy due to the volume difference between the unit cell used in the simulations and the same gas at standard state was determined using the following ideal gas free energy difference:

$$\Delta A_{\mathrm{corr}} = k_{\mathrm{B}} T \ln\left[\frac{V_0^N}{\Lambda^{3N} N!}\right] - k_{\mathrm{B}} T \ln\left[\frac{V_{\mathrm{cell}}^N}{\Lambda^{3N} N!}\right], \tag{S25}$$

Here, N is the number of gaseous hydrogen atoms or hydrogen molecules, V_0 is the volume (24.45

Table S 5. Nuclear quantum effects on the free energies of H_2O and H_3O^+ isolated in vacuum estimated as the difference between the quantum oscillator model and the harmonic oscillator model. The estimation using the experimental vibrational frequencies of solvated proton is also listed. Units of the free energy and vibrational frequencies are eV and cm^{-1}, respectively.

Species	Property	RPBE+D3	PBE0	PBE0+D3	Exp.
H_2O	ν_i	3831	4020	4020	
		3702	3886	3885	
		1592	1611	1611	
	$A_{q,vib}$	0.566	0.590	0.590	
	$A_{c,vib}$	0.087	0.089	0.089	
	A_{q-c}	0.479	0.502	0.502	
	ZPE	0.566	0.590	0.590	
H_3O^+	ν_i	3599	3761	3761	3020
		3598	3760	3760	
		3482	3650	3649	
		1647	1669	1669	1760
		1638	1652	1651	1250
		802	688	688	
	$A_{q,vib}$	0.916	0.941	0.941	0.374
	$A_{c,vib}$	0.157	0.157	0.157	0.074
	A_{q-c}	0.760	0.784	0.785	0.300
	ZPE	0.916	0.942	0.942	0.374
	Correction to $\alpha^0_{H^+}$	**0.281**	**0.283**	**0.283**	**0.300**
	ZPE[H_3O^+] − ZPE[H_2O]	0.350	0.351	0.351	0.374

L/mol) at the standard condition, Λ is the thermal de Broglie wavelength, and V_{cell} is the volume of the unit cell used in the computations.

S4. LOCAL POTENTIAL GAP $\Delta\phi$ BETWEEN SOLUTION AND VACUUM

Similar to our previous publication, the local potential gap $\Delta\phi$ between the solution and vacuum was computed using the difference of 1s levels of oxygen atoms of water molecules far from the solute in the bulk solution ($\varepsilon_{1s,bulk}$) and those at the middle of the water slab ($\varepsilon_{1s,slab}$) via Eq. (7) in the main text. To investigate the size effect, $\varepsilon_{1s,slab}$ was calculated using the RPBE+D3 functional on water slabs containing 96, 128, and 192 water molecules per unit cell, with respective dimensions of $11.4\times11.4\times44.2$ Å^3, $12.5\times12.5\times50$ Å^3 and $14.3\times14.3\times56.2$ Å^3, as shown in Fig. S10 (a)–(c). In these initial calculations, 2000 statistically independent snapshots were randomly selected from 10 trajectories generated by 100 ps NVT-ensemble MD simulations at 298 K using the MLFF. Additionally, we analyzed the orientation distribution of interfacial water dipole vectors in slabs with 128 and 1024 water molecules, using the MLFF, as reported in our previous study [7]. After confirming minimal size effects on $\Delta\phi$ (see Fig. S11) and on the orientation distribution [7], we selected the 128-water slab, which is computationally feasible for use with hybrid functionals. For the 128-water slab, we added 1000 statistically independent snapshots obtained from an additional five 100 ps NVT-ensemble MD simulation trajectories at 298 K using the MLFF. FP calculations employing the RPBE+D3 functional were then performed on these 3000 total snapshots. The value of $\varepsilon_{1s,slab}$ was determined as the average of 1s levels of the oxygen atoms in the shaded region in Fig. S10. Further details of the computational parameters are described in our previous publication. As noted in the main text, the TI simulation was performed using the average of $\Delta\phi$ for the solutions with and without a proton and an electron. The solutions were modeled by the unit cells shown in Fig. S1. For each system, 500 snapshots were selected from a trajectory of 100 ps NVT-ensemble MD simulation at 298 K employing the MLFF, and FP calculations using the RPBE+D3 functional were performed on the selected snapshots. The value of $\varepsilon_{1s,bulk}$ for the bulk pristine water was determined as the average of 1s levels of all oxygen atoms, while the one for the bulk solution with the solute was calculated as the average of all oxygen atoms $L/2$ away from the solute shown as the shaded region in Fig. S10 (d). Here, L means the length of the side of the unit cell. Because the RPBE+D3 already gives the accurate local potential, the change in the oxygen 1s level with changing the functional to the non-local hybrid functional is small. The small deviation can be efficiently computed by averaging the differences over 30 configurations for each bulk and slab system. The results of $\Delta\phi$ are tabulated in Table S6.

Table S 6. The local potential difference $\Delta\phi$ (V) between the bulk aqueous solutions and vacuum. The error shows the standard deviation determined by the block averaging analysis. The values for the PBE0+D3 functional are the same as those for the PBE0 functional.

System	RPBE+D3	PBE0	HSE06	B3LYP
$64H_2O$	3.64±0.02	3.52±0.02	3.53±0.02	3.60±0.02
H^++$64H_2O$	3.71±0.02	3.59±0.02	3.60±0.02	3.68±0.02
V^{2+}+$64H_2O$	3.72±0.02	3.61±0.02	3.61±0.02	3.68±0.02
V^{3+}+$64H_2O$	3.79±0.02	3.68±0.02	3.68±0.02	3.75±0.02
Fe^{2+}+$64H_2O$	3.73±0.02	3.60±0.02	3.61±0.02	3.68±0.02
Fe^{3+}+$64H_2O$	3.79±0.02	3.68±0.02	3.68±0.02	3.76±0.02
Cu^++$64H_2O$	3.73±0.02	3.60±0.02	3.60±0.02	3.68±0.02
Cu^{2+}+$64H_2O$	3.73±0.02	3.61±0.02	3.61±0.02	3.69±0.02
Ru^{2+}+$64H_2O$	3.79±0.02	3.68±0.02	3.68±0.02	3.75±0.02
Ru^{3+}+$64H_2O$	3.85±0.02	3.74±0.02	3.75±0.02	3.82±0.02
Ag^++$64H_2O$	3.76±0.02	3.64±0.02	3.63±0.02	3.72±0.02
Ag^{2+}+$64H_2O$	3.79±0.02	3.69±0.02	3.67±0.02	3.75±0.02
O_2^-+$62H_2O$	3.65±0.02	3.53±0.02	3.54±0.02	3.61±0.02
O_2+$62H_2O$	3.65±0.02	3.54±0.02	3.54±0.02	3.61±0.02

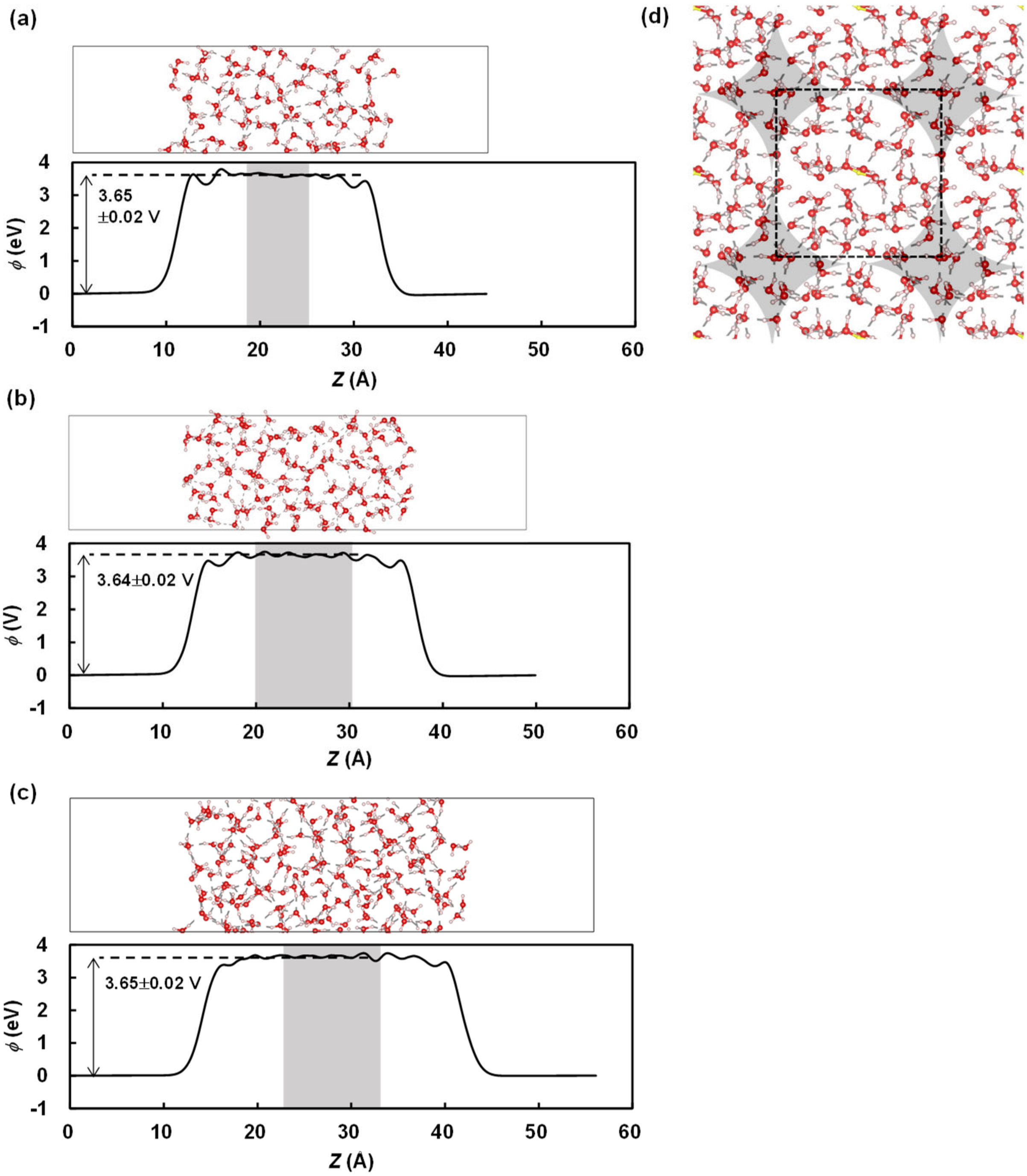

Figure S 10. Models used to compute $\Delta\phi$. Water slabs consisting of 96, 128 and 192 water molecules per unit cell [(a), (b) and (c), respectively] and bulk solution consisting of one proton and 64 water molecules per unit cell [(d)]. Averages of 1s levels of oxygen atoms in the shaded regions are used as $\varepsilon_{1s,slab}$ and $\varepsilon_{1s,bulk}$. The black line in the graph (a) to (c) shows the local potential profile.

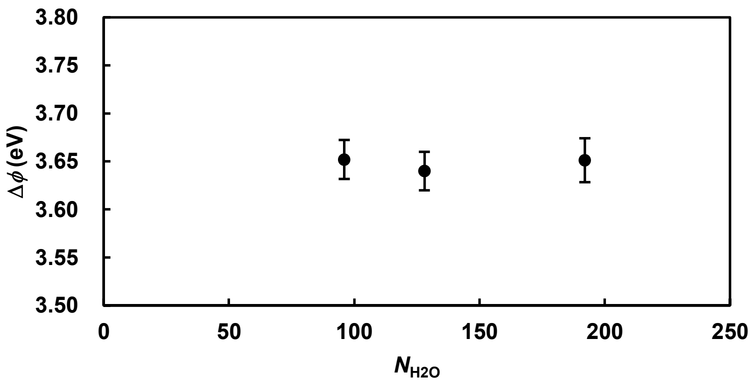

Figure S 11. $\Delta\phi$ as a function of the number of water molecules N_{H_2O} per unit cell of the slab.

[1] Jörg Behler and Michele Parrinello. Generalized neural-network representation of high-dimensional potential-energy surfaces. *Phys. Rev. Lett.*, 98:146401, 2007.

[2] Albert P. Bartók, Mike C. Payne, Risi Kondor, and Gábor Csányi. Gaussian approximation potentials: The accuracy of quantum mechanics, without the electrons. *Phys. Rev. Lett.*, 104:136403, 2010.

[3] Ryosuke Jinnouchi, Ferenc Karsai, and Georg Kresse. On-the-fly machine learning force field generation: Application to melting points. *Phys. Rev. B*, 100:014105, 2019.

[4] Albert P. Bartók, Risi Kondor, and Gábor Csányi. On representing chemical environments. *Phys. Rev. B*, 87:184115, May 2013.

[5] Ryosuke Jinnouchi, Ferenc Karsai, Carla Verdi, Ryoji Asahi, and Georg Kresse. Descriptors representing two- and three-body atomic distributions and their effects on the accuracy of machine-learned inter-atomic potentials. *The Journal of Chemical Physics*, 152(23):234102, 2020.

[6] Ryosuke Jinnouchi, Saori Minami, Ferenc Karsai, Carla Verdi, and Georg Kresse. Proton transport in perfluorinated ionomer simulated by machine-learned interatomic potential. *The Journal of Physical Chemistry Letters*, 14(14):3581–3588, Apr 2023.

[7] Ryosuke Jinnouchi, Ferenc Karsai, and Georg Kresse. Machine learning-aided first-principles calculations of redox potentials. *npj Computational Materials*, 10(1):107, May 2024.

[8] Feng Wang and Jun Cheng. Automated workflow for computation of redox potentials, acidity constants, and solvation free energies accelerated by machine learning. *The Journal of Chemical Physics*, 157(2):024103, 07 2022.

[9] Feng Wang, Zebing Ma, and Jun Cheng. Accelerating computation of acidity constants and redox potentials for aqueous organic redox flow batteries by machine learning potential-based molecular dynamics. *Journal of the American Chemical Society*, 146(21):14566–14575, May 2024.

[10] M. P. Allen and D. J. Tildesley. *Computer Simulation of Liquids*. 1987.

[11] Ryosuke Jinnouchi, Ferenc Karsai, and Georg Kresse. https://doi.org/10.5281/zenodo.14503891, 12 2024.

[12] Ryosuke Jinnouchi, Ferenc Karsai, Carla Verdi, and Georg Kresse. First-principles hydration free energies of oxygenated species at water–platinum interfaces. *The Journal of Chemical Physics*, 154(9):094107, 03 2021.

[13] Florian Dorner, Zoran Sukurma, Christoph Dellago, and Georg Kresse. Melting Si: Beyond density functional theory. *Phys. Rev. Lett.*, 121:195701, Nov 2018.

[14] Ryosuke Jinnouchi, Ferenc Karsai, and Georg Kresse. Making free-energy calculations routine: Combining first principles with machine learning. *Phys. Rev. B*, 101:060201, Feb 2020.

[15] Francesco Ambrosio, Giacomo Miceli, and Alfredo Pasquarello. Redox levels in aqueous solution: Effect of van der Waals interactions and hybrid functionals. *The Journal of Chemical Physics*, 143(24):244508, 12 2015.

[16] Francesco Ambrosio, Zhendong Guo, and Alfredo Pasquarello. Absolute energy levels of liquid water. *The Journal of Physical Chemistry Letters*, 9(12):3212–3216, Jun 2018.

[17] Jeongho Kim, Udo W. Schmitt, Julie A. Gruetzmacher, Gregory A. Voth, and Norbert E. Scherer. The vibrational spectrum of the hydrated proton: Comparison of experiment, simulation, and normal mode analysis. *The Journal of Chemical Physics*, 116(2):737–746, 01 2002.

[18] Jun Cheng, Marialore Sulpizi, and Michiel Sprik. Redox potentials and pKa for benzoquinone from density functional theory based molecular dynamics. *The Journal of Chemical Physics*, 131(15):154504, 10 2009.

[19] Francesca Costanzo, Marialore Sulpizi, Raffaele Guido Della Valle, and Michiel Sprik. The oxidation of tyrosine and tryptophan studied by a molecular dynamics normal hydrogen electrode. *The Journal of Chemical Physics*, 134(24):244508, 06 2011.
